# Presbyopia: An outstanding and global opportunity for early detection of pre-frailty and frailty states

**DOI:** 10.3389/fmed.2022.968262

**Published:** 2022-10-04

**Authors:** Almudena Crooke, Irene Martínez-Alberquilla, David Madrid-Costa, Javier Ruiz-Alcocer

**Affiliations:** ^1^Department of Biochemistry and Molecular Biology, Faculty of Optics and Optometry, Universidad Complutense de Madrid, Madrid, Spain; ^2^Clinical and Experimental Eye Research Group, Faculty of Optics and Optometry, Universidad Complutense de Madrid, Madrid, Spain; ^3^Department of Optometry and Vision, Faculty of Optics and Optometry, Universidad Complutense de Madrid, Madrid, Spain

**Keywords:** presbyopia, frailty, age-related eye diseases, tear, pre-frailty

## Introduction

According to the World Population Prospects 2022 of the United Nations, the world's population will rise over the next decades (e.g., by 6.3 and 21% from 2022 to 2030 and 2050, respectively) (1). Likewise, the population of older persons is increasing (1). Indeed, the United Nations expects that the share of the global population aged 65 years and above will rise by 6% between 2022 and 2050 (1). Consequently, age-related diseases, including eye ones, are expected to be very prevalent (2–4). For this reason, population aging is a crucial demographic issue with a growing global impact on all socioeconomic areas (4). This fact has provoked different global initiatives like the one declared by the United Nations in 2020, called the Decade of Healthy Aging 2021–2030, whose aim is to promote and maintain the wellbeing of older adults (5). In this context, the Lancet Global Health Commission on Global Eye Health argues that eye health should be part of such strategies focused on achieving universal healthy aging (6). Indeed, vision impairment and blindness affect multiple functional domains (physical, cognitive, psychological, social) and overall quality of life and wellbeing (6). Now, 510 million people have impaired vision, and 43.3 million are blind (7). Among these people, older adults present with a moderate/severe vision impairment and blindness prevalence of 112 cases and 18.5 cases per 1,000 people, respectively (7). Age-related eye diseases (e.g., cataracts, glaucoma, age-related macular degeneration, and diabetic retinopathy) are the leading global causes of visual impairment and blindness (6, 8). Hence, as the population ages, an increase in those numbers is expected, and by 2050 866 million and 61.0 million people will have moderate/severe vision impairment and be blind, respectively (7). Consequently, this global eye health initiative urges, like healthy aging initiatives, an in-depth understanding of the aging process and its associated diseases for preventing or delaying age-related eye conditions (6, 9).

## Age-related eye changes: Presbyopia

The aging process involves a progressive decline of all organ-specific functions, including eye ones. Like the rest of the body, the eye undergoes age-triggered changes that alter its structures, impairing its physiological functions [(10) review eye changes critical for the onset of age-related eye diseases]. One of these impaired functions is the accommodation process. This process, which allows focusing on near objects, occurs by the harmonized action of the ciliary muscle and the zonule fibers which hold the lens in place. Aging triggers changes in these structures involved in accommodation, leading to the gradual inability of the eye to focus (11, 12). This physiological event called presbyopia starts to express itself at around 40 years and affects 100% of the population by age 50 (13, 14). The tell-tale symptom of presbyopia is blurred vision while reading, sewing, using a mobile phone, tablet, computer, or doing anything that requires intermediate and near vision (5). Furthermore, presbyopia may present with eyestrain and headaches after reading or doing close-up work (5). Therefore, it negatively impacts the individuals' quality of life, urging them to seek a solution from an eye specialist (15–17).

## Frailty and eye

Frailty is an age-related syndrome that implies changes at all physiological levels, leading to a state of vulnerability, which could facilitate age-related disease onsets (18). A recent systematic review has shown that the overall prevalence of frailty and pre-frailty among individuals aged 50 years and older varies between 12–24% and 46–49%, respectively (19). Likewise, another systematic review has revealed that the incidence of frailty and pre-frailty among older adults is 43.4 and 150.6 new cases per 1,000 person-years, respectively (20).

Several recent studies have identified pre-frailty/frailty signs in middle-aged adults (40–50 years old) (21–23). Even more notable is that this frailty/pre-frailty condition is associated with multimorbidity and mortality in UK older/middle-aged participants of a prospective analysis (22). So, early detection of this condition could allow rapid implantation of measures that prevent or delay these poor health outcomes (22). Unfortunately, its multiple signs and symptoms (often non-specific) and the limited knowledge of its underlying molecular mechanisms (mainly in middle-aged adults) have hindered its early diagnosis (18).

The World Health Organization (WHO) has introduced the concept of intrinsic capacity (IC) (i.e., the combination of all the individual's physical and mental capacities) as a crucial component of healthy aging (24). It has also provided recommendations and tools to manage IC decline at the community level and primary care level, assuring integrated care for older adults (ICOPE) (25, 26).

According to the WHO ICOPE guidelines, vision is a critical component of IC (26). A simple eye chart permits the measurement of visual capacity, and distance acuity worse than 6/18 implies moderate vision impairment that needs further diagnostic assessment (26).

In one US study with 2,705 older adults, individuals with near vision impairment were more likely to be pre-frail and frail than those without visual loss (27). This result suggests an association between vision impairment, which promotes IC decline, and frailty (27). In this sense, some prospective studies have found that IC decline overlaps with frailty syndrome and can predict poor health outcomes in older adults (28–30).

Another UK prospective study with 493,737 middle-aged adults and older adults showed that individuals with glaucoma had a high prevalence of pre-frailty and frailty conditions (41.2 and 5%, respectively) (22). Equally, Wang et al. have found in a prospective China population-based study that this disease is associated with 10-year mortality (31).

The previously mentioned study by Hanlon et al. of middle-aged and older adults also revealed that patients with diabetes presented higher pre-frailty and frailty prevalence (54.8 and 13%, respectively) than glaucomatous ones (22). Besides, a systematic review has proved an association between diabetic retinopathy (DR, a major microvascular diabetes complication) and poor psychosocial functioning, affecting the quality of life of these patients (8, 32, 33). Likewise, a retrospective cohort study with 477 participants found that both frailty and diabetic microvascular complications can predict adverse clinical outcomes (e.g., emergency hospitalizations, institutionalization in a long-term care facility, falls, fractures, and death) in diabetic older adults (34).

Although the prevalence of pre-frailty/frailty in individuals with age-related macular degeneration (AMD) is yet unknown, Zhu et al. have suggested in a prospective study that late AMD is a biomarker of frailty syndrome (35). These authors argued that the poor survival observed in late AMD participants could be due to age- and frailty-related systemic comorbidities that coexist and share underlying molecular mechanisms with AMD (35). Moreover, several prospective studies have demonstrated that patients with AMD present a higher risk of falls and fear of falling, which leads to a decreased quality of life and disability (36–38). This fear of falling also has been observed in glaucomatous patients using the same validated questionnaire (the University of Illinois at Chicago Fear of Falling Questionnaire) (39). Some systematic reviews have also demonstrated an association between the fear of falling and poor quality of life with frailty (40, 41). Therefore, all these data seem to connect AMD and frailty.

A prospective study performed with age-related cataract patients found that they have poor survival rates, suggesting that cataracts, the most important cause of visual impairment and blindness, are also a biomarker of frailty (42). This study confirmed a previous cohort study that had shown the association between age-related cataracts and some measures of frailty independent of visual acuity and systemic comorbidities (43).

Villani et al. have built an ocular surface frailty index (OSFI) and tested *via* a longitudinal study its capacity to identify frail-ocular surfaces among patients who underwent cataract surgery (44). Consequently, these authors propose OSFI as a tool to predict patients with a high risk of post-surgical development of dry eye disease (DED) (44). This disease is also an age-related condition of the ocular surface that represents a growing problem with a substantial negative impact on the quality of life and global economy (45, 46).

To support this subsection, we searched in PubMed for the combination of the words: “frailty” and “eye” or one of the four age-related eye diseases leading causes of visual impairment/blindness: “cataracts”, “glaucoma”, “age-related macular degeneration”, and “diabetic retinopathy”. As we only aimed to summarize knowledge concerning this subsection's topic, among all articles found, we selected those most recent and focused on our point of view.

## Eye as a source of diagnostic biomarkers

The eye and especially the tear film have become, in recent years, the target for researchers of being an outstanding source of biomarkers for the diagnosis of both ocular and systemic diseases such as dry eye, Sjogren's syndrome, keratoconus, cancer, and COVID-19 (47–52). The main reason for this is that tears are the most accessible corporal fluid, and collecting them is easier, faster, and less invasive than the collection methods of other fluids (53).

Tear film covers the external ocular surface and consists of an inner mucous/aqueous and an external lipid phase, presenting a great diversity of macromolecules that undergo measurable changes in pathological conditions (54–58). Among these conditions are age-related diseases, including eye diseases. So, tears have provided several potential biomarkers for cataracts, glaucoma, AMD, DR, Alzheimer's, and Parkinson's diseases (57, 59–63). Conversely, the presence of specific frailty biomarkers in tears is unknown.

## Discussion

Age is a driving factor for frailty and age-related diseases, sharing underlying molecular mechanisms (64–66). According to the population aging and life expectancy prospects, these conditions, including eye-related ones, will be very prevalent (1, 2, 64). Age-related eye diseases are the world leading causes of visual impairment and blindness (6, 8). Hence, as the population ages, a growing number of visually impaired and blind people is expected, which will have an enormous humanistic and economic impact (8). These visual problems decrease the IC and quality of life of those affected by it and are associated with frailty syndrome (6–8, 27). Moreover, age-related eye diseases coexist with pre-frailty/frailty syndrome and are potential biomarkers of frailty and predictors of poor health outcomes (8, 22, 31, 34, 35, 42). These data reflect the crucial role of visual performance in achieving healthy aging (6). In this context, future studies should explore the validity of including new visual function-related tests in primary care for the integrated attention of older adults (6, 37, 39–41, 67). Indeed, some vision experts claim to perform the contrast sensitivity test to evaluate the fear of falling (a marker of poor quality of life, disability, and frailty) because it is a better predictor of this fear than the visual acuity test (37). Moreover, a recent cross-sectional study has found that poor contrast sensitivity is associated with frailty (68). Likewise, the older adults' health programs could include questionnaires to measure fear of falling and quality of life previously validated in patients with age-related eye diseases (37, 39, 67).

The pivotal role of visual performance in achieving healthy aging also urges research in the diagnosis and treatment of age-related eye disorders fields to implement new global preventive and therapeutic strategies against those diseases (6).

Frailty syndrome, a geriatrician's high-priority theme, has become an emerging target of gerontologists. They have found that this disorder that predisposes a person to age-related disease onsets is present in older and middle-aged adults and is associated with mortality, particularly in individuals with multi-morbidity (18, 21–23, 69). Given that a rapid intervention can reverse the condition, thus preventing its poor health outcome, gerontologists recommend screening frailty biomarkers in middle-aged adults (from the fourth decade of life onwards) (21–23, 69).

In this life period, presbyopia can also occur. This physiological process gradually reduces the ability of the eye to focus at different distances, impacting individuals without and with refractive errors (e.g., myopia, hyperopia, or astigmatism) who start to feel presbyopia symptoms from 40–50 years (12, 14). Because of these presbyopic symptoms, the entire population of middle-aged adults will visit eye care professionals seeking a solution. Probably, no other biomedical professionals attend to the whole population of middle-aged adults. This fact is remarkable because, as we have commented above, screening frailty biomarkers in middle-aged adults is critical for timely interventions to prevent age-related diseases and mortality.

Some data support the concept of age-related eye diseases as biomarkers of frailty phenotype and predictors of poor health outcomes (8, 22, 27, 31, 34, 35, 42, 67). Equally, data back the concept of the eye and its tear as a source of diagnostic biomarkers of ocular and systemic diseases, including age-related ones (57, 59–63). Thus, it would not be surprising that tears would contain frailty biomarkers. As any eye practitioner can easily collect tears, screening for frailty biomarkers from tears of presbyopic subjects may represent an outstanding opportunity for early detection of pre-frailty and frailty states, allowing timely intervention and thus preventing poor clinical outcomes.

Molecular mechanisms of frailty could also arise at the eye level, as occur with aging and age-related diseases (65). Indeed, a systematic review has revealed recently that frailty mechanisms occur in oral tissues (70). In this sense, the prospective study of Villani et al. has suggested the existence of frailty underlying mechanisms at the ocular surface of individuals who undergo cataract surgery (44). Thus, a future screening of frailty biomarkers from tears of presbyopic subjects could be a simple method of studying possible ocular surface frailty mechanisms and how they could link to the processes that occur in the rest of the eye and body. The understanding of these mechanisms could provide new biomarkers, helping delay age-related diseases onsets, including eye ones.

In summary, this article aims to show that theoretically, it is possible to perform a simple and large-scale frailty screening of middle-aged adults' tears, taking advantage of the unavoidable visit of presbyopic individuals to eye care professionals looking for a solution to their symptoms. The previous search for frailty biomarkers taken from tears of presbyopic people would allow this screening and thus timely interventions, delaying age-related diseases onsets and mortality.

We have confidence in the value of the tears of presbyopic people as an easy means to identify frail/pre-frail individuals, validate frailty biomarkers candidates, and study local frailty molecular mechanisms, which will provide new biomarkers.

## Author contributions

AC, DM-C, and JR-A contributed to the research conception. AC, JR-A, and IM-A contributed to the literature review. AC and JR-A contributed to the manuscript writing. All authors contributed to the article and approved the submitted version.

## Funding

This work has received funding from the European Union's Horizon 2020 research and innovation programme under grant agreement No. 956274. IM-A holds a predoctoral fellowship from Universidad Complutense de Madrid and Banco Santander, Spain (CT63/19-CT64/19).

## Conflict of interest

The authors declare that the research was conducted in the absence of any commercial or financial relationships that could be construed as a potential conflict of interest.

## Publisher's note

All claims expressed in this article are solely those of the authors and do not necessarily represent those of their affiliated organizations, or those of the publisher, the editors and the reviewers. Any product that may be evaluated in this article, or claim that may be made by its manufacturer, is not guaranteed or endorsed by the publisher.
